# Sharing the British National Health Service around the world: a self-interested perspective

**DOI:** 10.1186/1744-8603-9-51

**Published:** 2013-10-25

**Authors:** Kalipso Chalkidou, Jeanette Vega

**Affiliations:** 1 NICE International, National Institute for Health and Clinical Excellence, 10 Spring Gardens, SW1A 2BU London, UK; 2 Health, The Rockefeller Foundation, 420 5th Ave, New York, NY 10018, USA

**Keywords:** National Health Service, Universal Healthcare Coverage, Global health diplomacy

## Abstract

As the UK reiterates its commitment to protecting and growing its development aid budget amidst an adverse economic environment for the UK and Europe, we discuss the potential to use the country’s National Health Service (NHS) model as a vehicle for promoting the country’s economic as well as global health diplomacy and development priorities, through a coordinated cross-government plan of action. With the country’s Prime Minister serving as a co-chair of the UN post-2015 development agenda panel,^a^ this is a unique opportunity for the UK to put forward its health system architecture as a highly applicable and well-tested model for providing access to efficient and cost-effective care, with minimal financial hardship. Arguably, such a model tailored to the needs of specific countries could consequently lead to commercial opportunities for UK plc. in areas such as consulting, training, education and healthcare products. Finally, this approach would be consistent with the current thinking on the evolving role of UK aid, especially in the case of emerging powers such as India, where the focus has shifted from aid to investment in technical assistance and cooperation as a means of boosting bilateral business and trade.

## The NHS as a trade, foreign and global development policy tool

In December 2012, the United Nations’ General Assembly voted in favour of a resolution for Universal Health Coverage (UHC) [1], firmly placing the UHC movement at the centre of the discussions on what takes the place of the Millennium Development Goal framework [2] come 2015. A few days later, the US State Department set up a new office of Global Health Diplomacy and appointed its first ever Global Health Ambassador to: “…work with ambassadors to build political will in countries, in pursuit of sustainable health systems without barriers to care” [3].

As the UK reiterates its commitment to reaching the 0.7% GDP target for its development assistance, despite the adverse economic environment for the UK and Europe, we believe that the NHS can serve as a vehicle for promoting the UK’s trade, health diplomacy and global development priorities, through a coordinated, strategic long-term plan of action, cutting across government departments. There are major commercial benefits of 'exporting’ the NHS model stemming from an enhanced UK brand, stronger governance and regulation and ability to influence the structures of some of the largest and fastest growing markets in the world, as well as from direct commercial opportunities for UK universities, hospitals and consultancies.

Furthermore, there are also major philanthropic and foreign policy benefits of such a coordinated effort for sharing the (good and bad) lessons of a National Health Service. These include helping address the Department for International Development objective of reducing poverty through establishing fair and equitable health systems which protect individuals from financial catastrophe and offer care to those in need. At a time when aid initiatives in emerging markets are being scaled down, NHS know-how sharing can lead to the development of Southern centres of excellence for supporting the transition towards UHC, both technically and in terms of process.

**Figure 1:**
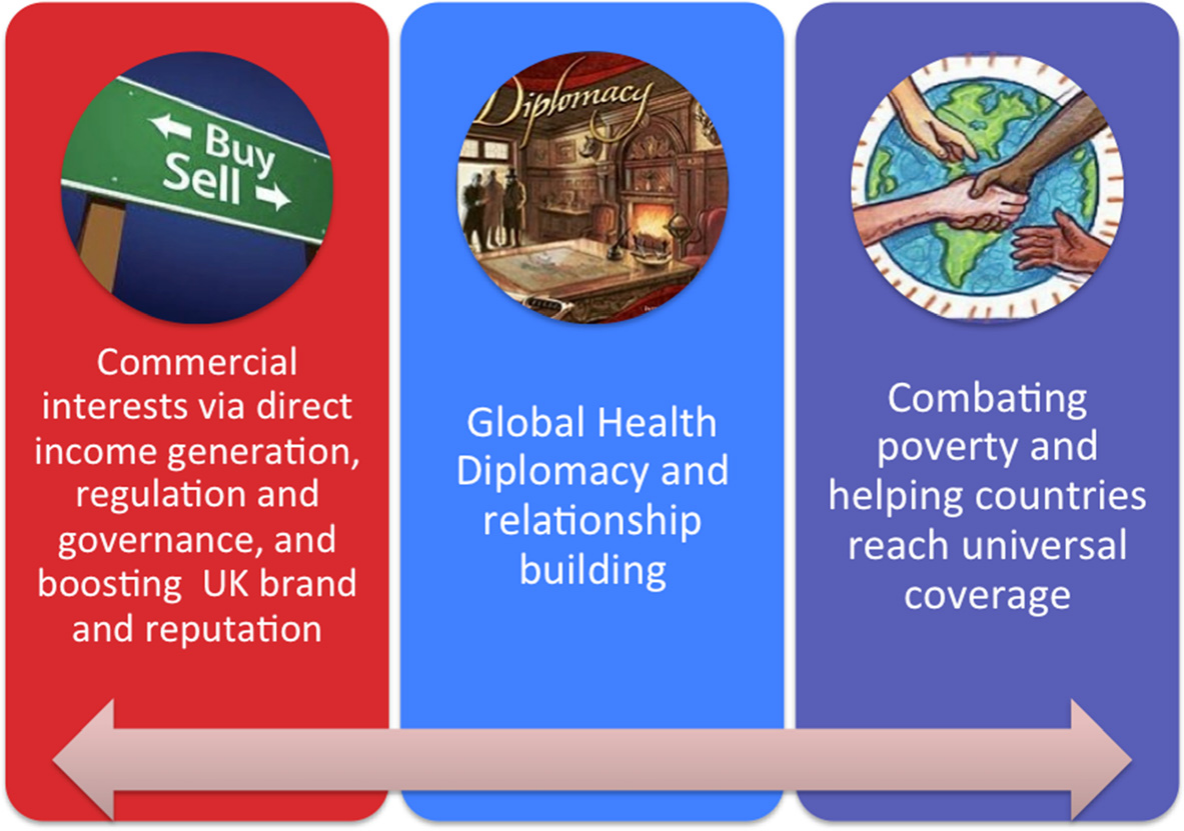
**Getting the balance right.** The National Health Service can serve as a means of meeting the triple objective of poverty eradication and development through universal coverage; global health diplomacy and relationship building; and, improving trade and the UK’s commercial interests overseas.

Added to the philanthropic objectives, there are foreign policy and diplomacy ones: global health diplomacy is a growing field, with the US having recently appointed a Global Health Ambassador accountable to the country’s Secretary of State. The UK Government can benefit from a more proactive approach to relationship building through sharing sought after experiences of the NHS (see Figure 1).

In actively promoting such international engagement, the NHS stands to learn from others and benefit as it is entering an era of significant underfunding.^b^

## Moving towards universal health coverage: the global momentum

The World Health Organisation defines Universal Health Coverage, as: “…*securing access for all to appropriate promotive*, *preventive*, *curative and rehabilitative services at an affordable cost*” [4]. As such, UHC incorporates both protection from catastrophic costs [5] as well as access to good quality care for all those who need it [6].

The global movement for UHC is gaining momentum. In their first ever Health Summit, held in Beijing in 2011, the Health Ministers from all 5 BRICS countries reaffirmed their commitment to working together and with other countries to ensure equitable access to “medicines and health commodities”.^c^ National health reforms in the co-called emerging powers, are on-going. China is rolling out an innovative multi-billion health insurance scheme to cover its 850 million rural citizens [7]. In its 12th Plan, India recently announced its commitment to achieve UHC by doubling the public funds spent on healthcare to over 2% of the country’s GDP [8,9]. Turkey is about to declare (and celebrate) Universal Coverage in 2013, and Mexico [10], Chile [11] and Thailand [12] are major success stories in offering healthcare and financial protection from health spending, to all of their citizens. South Africa, Indonesia, Ghana, the Philippines, are some of the many low and middle income countries whose governments are now legislating and mobilising resources to reach the UHC goal within the coming years [13]. The United States of America is also working toward expanding access during the second Obama Administration [14].

## The British National Health Service: focus on quality, efficiency and access…

Set up immediately after the 2nd World War, and built on the principles of equity and solidarity, the British National Health Service is the largest and one of the oldest single-payer publicly funded national healthcare systems in the world. For over 60 years, the NHS has remained true to its core principles of comprehensiveness and universality, which, amongst other attributes, such as minimal out-of-pocket contributions and no or nominal fees at the point of care, make it a successful model to provide universal health coverage to the UK citizens, most remarkably as the UK has just below average OECD levels of spending on healthcare. As it stands today, the NHS is defined by a set of attributes, determined to an extent by contextual factors such as custom, feasibility, resources and prevailing ideology, some of which (such as the ownership of the delivery agents) have changed over the years.

However, recent reforms may have, according to some, challenged some of the NHS’s core attributes [15]. Changes brought about by the Coalition government regarding the way care is purchased (GPs now have responsibility for the bulk of NHSs budget) and even delivered with an increasing role for private providers and a push towards competition between institutions, may seem to be undermining some of the core founding principles of the NHS and have fuelled a bitter and highly polarised debate.^d^ Most importantly, the ongoing recession, has meant that NHS, though its budget has been ring-fenced, is faced with significant efficiency savings, making restructuring amidst a shrinking (given the growing demand) budget, all the more challenging [16].

Interestingly, the reforms have triggered the interest of overseas policy makers who want to understand the arguments on both sides and the government’s rationale, and assess the relevance of the reforms to their own setting. Overall, and despite recent changes, in the eyes of many LMIC governments and citizens, the NHS and its core and core attributes, represent a successful, British application of the UHC model.

The UK NHS scores consistently high on international benchmarking comparisons of health system performance across developed countries [17,18] especially on equity, integration, macro efficiency and cost effectiveness.

In addition, almost 65 years on, the NHS enjoys high levels of public satisfaction (~60% in 2011 and ~70% in 2010 [19]) and broad cross-party support.

Furthermore, the UK has a strong reputation in basic science and clinical research; an internationally renowned tradition in professional training and clinical governance; and a track record in evidence-informed policy and practice. Arguably, the NHS is the backbone of this success. With its Royal Colleges and medical schools, its standard-setting institutions, such as the National Institute for Health and Care Excellence (NICE), its strong commitment to research through its research councils and the National Institute for Health Research, its recent policy reforms in areas such as governance (e.g. Foundation Trusts, NHS Commissioning Board) and provider payment (e.g. Payment by Results, Quality and Outcomes Framework, Clinical Commissioning Groups), and its primary care-centred approach to prevention and management, the NHS regularly attracts interest from policy-makers and professionals from around the world.

### …and a (surviving) commitment to equity

Out of all the attributes set out above and in Table 1, the ones that differentiate the NHS from other systems that have also reached universality of coverage, and which are perhaps more relevant to the UHC movement, are its *equity in financing* (the way resources are raised and pooled and how this affects the most vulnerable groups in the society) through progressive general taxation and its *equity in resource allocation* (the way resources are managed and shared out between different geographical regions, population groups and then invested in, through a purchasing function, specific technologies and services).

**Table 1:** **Core attributes of the National Health Service**, **reflecting one possible** (**and fairly successful**) **implementation of a Universal Healthcare Coverage system**

**Attribute**	**Description**
*Comprehensiveness*	Cradle-to-grave system, covering effective and cost-effective health care, public health and complementary social care. In the 'ultimate’ case, 'comprehensiveness’ embraces all health determinants from pre-natal to the grave.
*Universality*	All residents of the UK (citizens, legal and illegal immigrants) are covered.
*User fees*	Care is free at the point of use
*Funding source*	Progressive general taxation funding
*Resource allocation*	Centralised resource allocation formula which takes account of need, deprivation, market forces
*Purchasing*/*commissioning*	NICE explicitly accounts for the opportunity cost of new investment decisions in services and technologies
*Ownership of healthcare delivery agents*	Mix of public, private and third sector, all under contractual arrangements to offer care to NHS patients based on fee schedule and quality standards
*Co*-*existence with private system*	Small (and shrinking) private market co-exists with the NHS but no opt-out of the NHS exists for British tax-payers

Providing 100% effective and equitable coverage at relatively low cost is perhaps what makes the NHS most attractive to developing country politicians, faced with the challenge of sustaining ever increasing costs in systems with relatively small formal sectors and weak administrative and information systems, hence making taxation a simple and attractive model. Indeed, Mexico, Brazil, Turkey and Thailand are UHC success stories, where taxation has been the major source of funding.

On resource allocation, the NHS has applied tools such as the Resource Allocation Working Party (RAWP) Formula, which since the mid 70s aims to allocate funds to local areas so that '…there would eventually be equal opportunity of access to health care for people at equal risk’. In the late 90s, the Labour government introduced another objective of resource allocation: 'to contribute to the reduction in avoidable health inequalities’ and whilst this has been down-weighted by the current Coalition government, it remains a driving principle of resource allocation [20]. In addition to the allocation formula which explicitly considers need, another policy intervention related to the purchasing of services, the National Institute for Health and Care Excellence (NICE) also emphasises the NHS’s commitment to taking account of the opportunity cost of every investment decision in healthcare. NICE aims to ensure that the NHS only invests in technologies and services that are proven to work for its population and that are good value for money. Equal access for equal need (in its clinical programmes) and an explicit objective to targeting disadvantaged groups (in its public health and possibly newly introduced social care programmes) are key driving principles for NICE [21,22].

## Growing global influence

Historically countries like Chile, as well as Cuba and Sri Lanka, were amongst the first to emulate the British model characterised by universal coverage, funded by general taxation, free at the point of service, and public ownership and/or regulation of health care delivery of services. Several others, such as Germany, Japan, and the Netherlands, introduced social insurance models based on prepaid contributions and mixed delivery of health care through public or private providers [23-25].

Free-market reforms in the 80s and 90s, often in the form of structural adjustment programmes advocated by donors, may have compromised the principles of access and solidarity. China is perhaps the biggest example of a whole country abandoning its communal model only to reinvent in the late 90s as the New Cooperative Medical Scheme and invest heavily in it since, as a means of covering the whole of its rural population [26]. The UK perhaps played a more active role in influencing this decision through a little-advertised 5-year DFID-sponsored programme, the Health Policy Support Project, which helped the Government of China decide, based on evidence from 8 countries, including the UK, to re-socialise its health financing system with an injection of $140 Billion of tax funding in 2009, with a growing annual tax-funded commitment to healthcare, every year since.^e^

As the UHC movement gains momentum, there has been a rapidly growing demand for technical support from many of the institutions that compound the NHS, including its National Institute for Health and Clinical Excellence [27], has been growing. Colombia launched in 2012, IETS, a priority setting institution modelled along the lines of the NHS’s NICE [28] to support priority setting across its insurance plans. The Government of India announced in its 12th Plan [9], its commitment to UHC along with its ambition to set up a NICE [29]. China, following almost 3 decades of market reforms, is now investing in a system with great government involvement and less out of pocket contributions, much closer to the British, including a Chinese version of NICE [7]. The influence of the NHS extends also to the international press and blog sites. Countries like China, Brazil and Chile, heavily featured and celebrated the British people’s affection of the NHS as reflected in the 2012 Olympic opening ceremony^f ^[30]. And whilst history and the political economy of the UK and of England in particular have determined the birth and evolution of the NHS and are highly context specific, countries share both values (solidarity, universality) and technical challenges (efficient financing and allocation), making cross country comparisons and know-how sharing, a popular and most likely useful activity.

Despite the burgeoning demand from countries moving towards UHC, sharing, through a single cross-government strategy, the UK system’s approach and know-how for building an integrated quality-driven national health system, has not been a government priority. There have been few government and third sector-sponsored initiatives over the past few years, aiming to promote some aspects of the NHS and the British healthcare industry. These include recently launched Healthcare UK, with an explicitly commercial short-term focus on catalysing business deals, the India-UK CEO’s Forum, with support from the Wellcome Trust for its health stream [31], and the DFID-sponsored Health Partnerships Scheme [32], unique in its NHS focus but relying on NHS staff volunteering their time and with an allocation of less than 0.06% of DFID’s annual budget. Perhaps the most ambitious, strategic and cross-cutting amongst these initiatives, the Department of Health’s Global Health Strategy, which includes all three, diplomatic, trade and development objectives, seems to lack the dedicated resources needed to deliver [33]. Some of these initiatives are further discussed, along with their advantages and weaknesses, in Table 2.

**Table 2:** **Recent initiatives by Her Majesty**’**s Government for promoting the NHS overseas**

**National policy or initiative**	**Brief description and challenges**	**Government or other sponsor**
Health Partnerships Scheme^o^	Aims “to support the development of health services in some of the world’s poorest countries” drawing on the experience of the NHS, academe and professional organisations.	Department for International Development – DFID; (NHS organisations and employees)
	*Relies largely on NHS* (*or individuals*) *volunteering their time*/*resources*: *challenging in current financial environment and may undermine longer*-*term sustainability of partnerships esp*. *in sub*-*Saharan Africa*.	
Health is Global^p^	An attempt to articulate a cross-government vision for global health, through an outcomes framework for global health (2011–2015) setting out set out how UK Government departments should work together coherently to improve health in the UK and overseas	Department of Health – DH; (DFID)
	*An independent review of the strategy highlighted lack of cross*-*government coordination*, *leadership and UK institutional champions and a lack of financial*[33].	
Global Development Partnerships Programme^q^	Aimed at ensuring coordination between DFID and other government departments and No 10, with an emphasis on DFID’s engagement with emerging powers and a view to catalyse South/South partnerships and boost the civic/donor responsibility of the BRICS countries.	DFID
Healthcare UK	New (Dec 2012) unit jointly sponsored and resourced by UKTI and DH and hosted by UKTI aimed at exporting UK expertise in healthcare (NHS and commercial), through a project-specific high priority country approach.	UK Trade and Industry/BIS; (DH)
	*No explicit relationship building or development focus*. *Fragmented and somewhat short*-*termist*	
NHS Global^r^	Predecessor to Healthcare UK, with a predominantly commercial focus aimed at supporting NHS-span businesses abroad	NHS (FCO; UKTI/BIS)
	*Was commercial remit and short term in its outlook* – *it has now been terminated*	
India – UK CEO Forum^s^	Established by the British and Indian Prime Ministers in 2010 to help achieve the two governments’ aim “to be ambitious in seeking to substantially increase trade and significantly increase investment between the UK and India”. Health identified as one of 4 key priorities [31].	Government of India – UK Government;
	*Lack of dedicated seed funding on the UK side* – *some initiatives such as primary care pilots in Kerala have been completed but unclear how they can now be scaled up*/*taken forward*.	For health: Wellcome Trust and No 10; (DH; UKTI; FCO; DFID)
Innovation, Health and Wealth^t^	NHS strategy (Dec 2011) for embracing and embedding innovation across the NHS, improving outcomes and driving growth for the UK. Sets out a requirement for NHS organisations to increase national and international activity” with a focus on commercial income generating opportunities.	NHS; (UKTI)

## The NHS as a catalyst for commercial opportunities

When it comes to the NHS, the UK has been putting increasing weight on the commercial, and often short-term and fragmented, side of global health, such as directly promoting British-made electronic medical record systems, medical tourism in the UK or abroad or large construction firms [34-37]. In the current economic downturn such an approach is perhaps understandable. However, commercial inroads are more likely to be achieved in an environment of broader engagement. Sharing the NHS thinking and overall architecture could be the first step in a broader relationship building strategy that will include but will not be driven by commercial interests. Promoting the NHS model, through a coherent cross-government strategy, as a highly applicable and well tested means for effectively financing a universal coverage system providing access to cost-effective care, could consequently lead to broad commercial opportunities for the UK in areas such as consulting, training, education and healthcare products. This approach is likely to benefit a number of UK sectors, including UK Universities (medical and nursing schools as well as epidemiology and statistical departments, schools of public policy and business administration), the Royal Colleges and the recently launched Academic Health Science Networks.

The medium-term benefits are also likely to be significant for the broader healthcare industry and third sectors involved in commissioning and provision, as well as infrastructure, telecom and IT, administration and outsourcing, and manufacturing of medical devices, pharmaceuticals and diagnostics. The UHC global agenda has opened up the prospects of significantly larger markets. Where the underpinning governance, purchasing and provision model are that of the British NHS, UK businesses are likely to benefit from familiar regulatory, financing and care delivery frameworks, including NICE-like systems for assessing value-for-money, and from a workforce which has been trained in and trusts the quality and evidence-informed care delivered by the NHS.

Furthermore, with innovation increasingly taking place outside the developed world and growing challenges to the intellectual property by developing country governments and their home-grown industry, strong partnerships which share the NHS as the preeminent model, are perhaps more likely (than aggressive litigation or restrictive Free Trade Agreements) to open channels of communication and generate potential for joint ventures, outward investment and win-win know-how transfer deals, such as the one between GSK and the Brazilian Government on the HPV and Dengue vaccines [38,39].

## The changing role of UK aid: engaging the emerging powers

In his Emerging Powers speech in June 2011, the then Secretary of State for Development described a changing world where relationships between the UK and emerging powers: “…*will become less rigid and more equal*” and where “*aid will be but one of our tools*. *We will trade in ideas and in expertise too*, *and we will broker political support and create coalitions to tackle specific issues*.”^g^

Know-how transfer in setting up and running a successful UHC system drawing on the experience of managers, clinicians, researchers, civil servants and policy makers working in or with the NHS, is less expensive and more appropriate than direct purchasing of commodities for all but the poorest of countries DFID operates in. For example, instead of purchasing pharmaceuticals on behalf of upper-middle income economies, the UK can help inform their governments’ decisions on formulary listing, procurement, pricing and reimbursement and evidence-based prescribing and use and build local technical and institutional capacity for making these decisions in the future. In a country like India, where 70% of all healthcare spending is out of pocket and over 70% of that goes towards purchasing pharmaceuticals, efficient, evidence-based procurement and use can release resources for expanding coverage to more people and investing in necessary technologies and services. The shift towards technical assistance focused on high priority areas including health and applying a cross-government approach, was announced by the Secretary of State for Development in her recent visit to India.^h^ In the same spirit, in February 2013, DFID announced the establishment of a tax capability unit within HMRC and further investment in DFID’s international growth centre, offering policy advice to governments on effective tax and growth policies, respectively.^i^ In a similar vein, though since scrapped, the previous administration set up the Centre for Progressive Health Financing to support countries achieve UHC [40].

Through forging functional partnerships, building trust and boosting governance and technical and administrative capacity, particularly in relation to emerging powers such as India and China, DFID can achieve its multiple objectives of:

• Catalysing South-South partnerships and boosting middle income countries’ ability to serve as value-adding responsible donors towards poorer states [41], also keenly interested in how China or Brazil have achieved UHC. This would also mean that the UK’s development agenda is sustained, even if DFID support is scaled down in the future.

• Facilitating exit strategies for DFID from providing expensive health sector inputs in middle income countries which should be replacing aid financing with domestic revenues, and optimising their spending through applying know-how from the UK as well as emerging Southern centres of knowledge (see earlier point).

• Delivering its poverty reduction agenda through supporting equitable and efficient UHC. Efficient resource allocation and scientifically robust and fair processes for setting priorities for what ought to be included in a basic benefits package, can serve as “equalisers” and means of domestic redistribution of resources and wealth within the so-called emerging economies, where the vast majority of world’s poor live [42].

• Ensuring the UK itself achieves demonstrable Value for Money, through promoting to key partners such as GAVI and the Global Fund, making significant investment on its behalf, the kind of economic evaluation the NHS, through NICE, applies before making major technology adoption decisions. Demonstrating value-for-money is an imperative, especially as DFID’s resources are being ring-fenced at a time of persistent economic recession back home.

## Aid for trade?

DFID is increasingly keen on using growth as a vehicle for development including through promoting British companies overseas.^j^ To achieve this, DFID acknowledges the importance of strong governance and regulation and is committed to helping build institutions to promote inclusive growth.^k^ In healthcare in particular, institutions like NICE that empower policy makers make wise investment decisions in an inclusive and procedurally fair fashion, are prerequisites for British industry, including large Pharma, expanding overseas in an ethical and beneficial for all, way.

This approach may also appeal to social impact investors, attracting capital for reinvesting into basic social objectives such as Universal Coverage and efficient healthcare systems through an authoritative peer-to-peer model of support which may develop into an income generating activity servicing large donors and multinationals as well as governments of emerging economies.

## Global Health Diplomacy: effective relationship-building

Diplomacy as a means of strengthening global health has been featuring high on the foreign policy agenda of the United States. The recent (Dec 2012) appointment of the first ever US Global Health Ambassador at the newly launched Office of Global Health Diplomacy to: “…*guide diplomatic efforts to advance the United States*’ *global health mission to improve and save lives and foster sustainability through a shared global responsibility*”,^l^ and the State Department’s sustained focus on global health policy, a form of “*smart*” power [43], helping build a “…*a safer*, *more secure world*”,^m^ are evidence of this. The UK can learn from the Americans as well as other major players such as Brazil [44] and Cuba,^n^ and use one of its most strategic assets, the NHS, in exerting global health diplomacy as a means for improving health and as a diplomatic end in itself.

Healthcare and affordable and high quality UHC feature highly in bilateral and multilateral fora (e.g. WHO/WHA; UN General Assembly) and High Level Economic Talks and Policy Dialogues, between countries, especially amongst the so-called emerging powers, which the UK is keen to influence. Indeed, recently signed Memoranda of Understanding between the UK and Mexico, Brazil, India and China all place particular emphasis on health systems and highlight the NHS as a resource for informing those countries’ own national reforms towards UHC.

A coordinated systems’ approach on the part of the British Government, including know-how transfer, sharing of information and data and exchanges and staff training programmes (for policy makers, clinicians and administrators) is likely to strengthen bilateral links at a small incremental cost to the British system. In turn, such engagement may offer potential openings for diplomatic dialogue in other, high priority areas for the UK, including trade, the environment and security.

## Learning from others

In an increasingly multicultural environment, the NHS can learn a lot about accessing socially vulnerable groups through engaging internationally. Poorer systems often develop coping mechanisms for managing under resource constraints, which may help the NHS adapt to its shrinking budget (e.g. more effective use of theatres, task shifting away from doctors and application of inexpensive technologies in the community setting) [45]. Though not as obvious perhaps a connection as with infectious disease outbreaks, addressing NCDs, enhancing access to affordable new technologies and reducing inequalities, are important and shared global challenges, potentially more effectively be tackled collaboratively, across rich and poorer countries. Experiences from the NHS’s volunteering programme have been most encouraging, leading the All Party Parliamentary Committee for global development to call for the “…*NHS England*, *the Department of Health and other national health bodies* [*to*] *reinforce the value and legitimacy of NHS involvement in global health by sustaining and extending successful policies*” [46]. We believe such meaningful two way learning can be one positive externality of “exporting” the NHS model, and requires investment on the part of the NHS in systematically identifying the lessons from developing countries’, and then adapting them to the UK setting and evaluating their impact. Most importantly, it requires a change in the NHS mentality, opening up to the possibilities of learning from colleagues operating in less developed and more resource constrained realities.

## Focusing on the how

Helping build functioning primary care systems and refocusing care away from tertiary hospitals and towards prevention and the outpatient setting; strengthening hospital governance and provider payment mechanisms; sharing experiences on pay for performance schemes and on quality standards; improving the efficiency of pharmaceutical listing decisions and helping train the next generation of healthcare professionals and ministry analysts, are only part of what UK technical assistance can achieve, all major building blocks of strong and sustainable UHC systems.

Such relationship-building through sharing the NHS model is also consistent with the British NHS’s public sector ethos and capabilities. Conversely, direct promotion of private goods is arguably better done by the private sector itself, which is also better at raising capital and taking risks when investing overseas.

## Conclusions

In an increasingly multicultural environment, the NHS can learn a lot through engaging internationally [45]. It may also be that, as the NHS learns more about the challenges other countries face, and how others view what has been achieved in the UK, the British policy makers come to value and perhaps better safeguard the most important aspects of their own system or become less prone to continuously restructuring it.

Using government funds to support 'public-public’ partnerships between the NHS and overseas healthcare systems is likely to improve diplomatic relations, spilling over to sectors beyond health and generating trade opportunities. This can be an inexpensive way to both respond to a well-articulated global demand and to build UK’s capacity better to share its expertise and experience with its overseas partners, as well as its ability to assess its own impact and learn in the process.

Lastly, under the stewardship of the UN, UHC is likely to play a central role in the post-MDG development world, beyond 2015. With the British Prime Minister having served as the co-chair of the post-MDG agenda panel, now is a unique opportunity for the UK Government to systematise its response to the demand for knowledge and technical assistance to learn from the NHS experience, whilst capitalising on the possibility of working across government sectors to address the UK’s foreign policy, trade and aid priorities.

This is the right time to commission a full economic analysis of both the costs and tangible and intangible (including those stemming from development and global health diplomacy) benefits of investing in promoting the NHS system, in a demand-driven way, versus the alternative of sole (and often short-termist and fragmented) commercial focus. This could include an options analysis of the governance and business arrangements of such an initiative, which could range from the NICE International low cost, opportunistic bottom-up approach to international engagements (see Table 3), to a more coordinated strategy, with a dedicated cross-government office, aligned with the new and emerging NHS infrastructure across England.

**Table 3:** **An example of a system**-**wide approach attracting cross**-**government support**[27]

**NICE International: Working across the British Government**	
NICE International, a non-profit, cost-recouping division of the National Institute for Health and Clinical Excellence, was set up by NICE’s Board in 2008, to support overseas governments with developing and applying the necessary technical and institutional capacity to improving the quality and efficiency of their healthcare systems.^a^ To achieve this, NICE International mobilises its own as well as broader NHS and non-NHS resources, front-line NHS professionals and UK Universities.	Traditionally relied on non-UK funding, from the World Bank, national governments of client countries, the European Commission, regional development banks such as the Inter American Development Bank, and, increasingly on philanthropic funders such as the Rockefeller Foundation and the Bill and Melinda Gates Foundation.
	During the second half of 2012, NICE International has received substantive funding support from the Department of International Development supplemented by funding from the Department of Health and small scale Foreign Office support mostly from FCO China, Brazil and the Philippines. NICE International has also been more directly involved in UKTI trade collaborative propositions, together with UK companies, in China and the Middle East
	NICE International is perhaps one example (with its strengths and weaknesses) for scaling up, across other parts of the NHS, cross-government working, driven by overseas governments’ requests for NHS expertise

## Endnotes

^a^UN Secretary-General Appoints High-level Panel on Post-2015 Development Agenda: http://www.un.org/sg/offthecuff/?nid=2455

^b^Special report: 'This can’t go on’ - NHS chiefs urge new debate on health service reforms, The Independent, July 2013, http://www.independent.co.uk/life-style/health-and-families/health-news/special-report-this-cant-go-on--nhs-chiefs-urge-new-debate-on-health-service-reforms-8701302.html

^c^For a transcript of the First BRICS Health Ministers Meeting in Beijing and the resulting Beijing Declaration of July 2011, see: http://www.cfr.org/global-health/brics-health-ministers-meeting----beijing-declaration/p25620

^d^See for example, from the BBC: Peers support government on NHS despite Labour 'privatisation’ warnings http://www.bbc.co.uk/news/uk-politics-22268417 April 2013 and from the Guardian, The NHS at 65: chaos, queues and mounting costs, July 2013 http://www.guardian.co.uk/commentisfree/2013/jul/05/nhs-65-chaos-queues-mounting-costs

^e^China Health Policy Support Programme, see: http://www.china.org.cn/english/2005/Oct/146196.htm and http://projects.dfid.gov.uk/project.aspx?Project=107715

^f^See for example tabloid coverage: http://www.dailymail.co.uk/news/article-2180227/London-2012-Olympics-Some-Americans-left-baffled-tribute-NHS-Mary-Poppins-Opening-Ceremony.html

^g^See Speech by Andrew Mitchell, International Development Secretary, at the Emerging Powers and the International Development Agenda at Chatham House on 15 February 2011: http://webarchive.nationalarchives.gov.uk/+/http://www.dfid.gov.uk/news/speeches-and-articles/2011/emerging-powers/

^h^http://www.dfid.gov.uk/News/Speeches-and-statements/2012/Justine-Greening-Update-on-aid-to-India/

^i^See written Ministerial Statement by the Secretary of State for International Development Justine Greening on aid to India in November 2012: https://www.gov.uk/government/policies/helping-developing-countries-economies-to-grow

^j^The government’s shift towards an aid for trade agenda, with an emphasis on growth as a mechanism for combatting poverty has been widely reported in the press. See for example: http://www.guardian.co.uk/global-development/2013/feb/07/justine-greenign-dfid-investment-africa-economic-growth and the Development Secretary’s original speech, setting out her priorities for UK aid in the coming years, hosted by ONE Campaign UK in February 2013: https://www.gov.uk/government/speeches/justine-greening-development-in-transition

^k^The government’s shift towards an aid for trade agenda, with an emphasis on growth as a mechanism for combatting poverty has been widely reported in the press. See for example: http://www.guardian.co.uk/global-development/2013/feb/07/justine-greenign-dfid-investment-africa-economic-growth and the Development Secretary’s original speech, setting out her priorities for UK aid in the coming years, hosted by ONE Campaign UK in February 2013: https://www.gov.uk/government/speeches/justine-greening-development-in-transition

^l^http://blogs.state.gov/index.php/site/entry/strengthening_global_health_by_elevating_diplomacy

^m^See US Secretary of State’s Hillary Clinton speech about the Obama administration’s Global Health Initiative to faculty and students at the Johns Hopkins School of Advanced International Studies in August 2010: What does global health have to do with foreign policy? Hillary Clinton: “everything”, http://www.globalhealtheurope.org/index.php?option=com_content&view=article&id=326:what-does-global-health-have-to-do-with-foreign-policy-hillary-clinton-qeverythingq&catid=85:opinion-pieces&Itemid=139

^n^See Medical Education Cooperation with Cuba: http://medicc.org/ns/ and here: http://blog.foreignpolicy.com/posts/2013/05/07/cuba_doctors_brazil_export_medical_diplomacy?wp_login_redirect=0

^o^http://www.thet.org/health-partnership-scheme

^p^https://www.gov.uk/government/publications/health-is-global-an-outcomes-framework-for-global-health-2011-15--2

^q^https://www.gov.uk/government/uploads/system/uploads/attachment_data/file/67419/glob-parts-dept-2011.pdf

^r^http://www.institute.nhs.uk/global/default/home.html

^s^http://ukindiaceoforum.com/

^t^http://www.institute.nhs.uk/images/documents/Innovation/Innovation%20Health%20and%20Wealth%20-%20accelerating%20adoption%20and%20diffusion%20in%20the%20NHS.pdf

## Competing interests

KC works for NICE, formerly an NHS organisation and, from April 2013, a UK Non Departmental Public Body.

## Authors’ contributions

Both authors have made substantial contributions to the conception, design and drafting of the paper. Both authors have read and approved the final manuscript.

## Authors’ information

KC is the founding director of NICE’s international division. NICE International receives funding from the Rockefeller Foundation.
